# Cancer Stem Cell-Like Circulating Tumor Cells Are Prognostic in Non-Small Cell Lung Cancer

**DOI:** 10.3390/jpm11111225

**Published:** 2021-11-18

**Authors:** Eva Obermayr, Nina Koppensteiner, Nicole Heinzl, Eva Schuster, Barbara Holzer, Hannah Fabikan, Christoph Weinlinger, Oliver Illini, Maximilian Hochmair, Robert Zeillinger

**Affiliations:** 1 Molecular Oncology Group, Department of Obstetrics and Gynecology, Comprehensive Cancer Center, Medical University of Vienna, Waehringer Guertel 18–20, 1090 Vienna, Austria; nina.koppensteiner@live.de (N.K.); nicole.heinzl@meduniwien.ac.at (N.H.); eva-maria.schuster@meduniwien.ac.at (E.S.); barbara.m.holzer@meduniwien.ac.at (B.H.); robert.zeillinger@meduniwien.ac.at (R.Z.); 2 Department of Respiratory and Critical Care Medicine, Karl Landsteiner Institute of Lung Research and Pulmonary Oncology, Klinik Floridsdorf, Bruenner Strasse 68, 1210 Vienna, Austria; hannah.fabikan@extern.gesundheitsverbund.at (H.F.); Christoph.Weinlinger@extern.gesundheitsverbund.at (C.W.); oliver.illini@gesundheitsverbund.at (O.I.); maximilian.hochmair@gesundheitsverbund.at (M.H.)

**Keywords:** non-small cell lung cancer, circulating tumor cells, cancer stem cell, microfluidics, gene expression analysis

## Abstract

Despite recent advances in the treatment of non-small cell lung cancer (NSCLC), less than 10% of patients survive the first five years when the disease has already spread at primary diagnosis. Methods: Blood samples were taken from 118 NSCLC patients at primary diagnosis or at progression of the disease before the start of a new treatment line and enriched for circulating tumor cells (CTCs) by microfluidic Parsortix™ (Angle plc, Guildford GU2 7AF, UK) technology. The gene expression of epithelial cancer stem cell (CSC), epithelial to mesenchymal (EMT), and lung-related markers was assessed by qPCR, and the association of each marker with overall survival (OS) was evaluated using log-rank tests. Results: EpCAM was the most prevalent transcript, with 53.7% positive samples at primary diagnosis and 25.6% at recurrence. EpCAM and CK19, as well as NANOG, PROM1, TERT, CDH5, FAM83A, and PTHLH transcripts, were associated with worse OS. However, only the CSC-specific NANOG and PROM1 were related to the outcome both at primary diagnosis (NANOG: HR 3.21, 95%CI 1.02–10.14, *p* = 0.016; PROM1: HR 4.23, 95% CI 0.65–27.56, *p* = 0.007) and disease progression (NANOG: HR 4.17, 95%CI 0.72–24.14, *p* = 0.025; PROM1: HR 4.77, 95% CI 0.29–78.94, *p* = 0.032). Conclusions: The present study further underlines the relevance of the molecular characterization of CTCs. Our multi-marker analysis highlighted the prognostic value of cancer stem cell-related transcripts at primary diagnosis and disease progression.

## 1. Introduction

Lung cancer is the second most common cancer and the leading cause of death worldwide. In 2020, 2.2 million new cases were estimated, accounting for 11.4% of all new cancer diagnoses [1]. The main histological type is non-small cell lung cancer (NSCLC), accounting for about 85% of all lung cancer cases. Despite recent advances in the treatment of NSCL, especially due to the identification of targetable driver mutations, the prognosis of patients remains poor, with a 5-year relative survival rate of 10–20% across all stages [2]. With localized disease, the 5-year survival rate is 63%; when the disease has already spread to distant organs, however, just 7% of patients are alive after 5 years [3].

A diagnosis of NSCLC is made based upon the pathologic evaluation of cytologic or histopathologic specimens. Staging NSCLC by computed tomography and other radiological procedures determines the appropriate therapy, and, when combined with the patient and tumor’s unique features, provides valuable prognostic information. The majority of patients are diagnosed at an advanced stage and, here, a systemic therapy is the standard of care. For patients with early-stage NSCLC, a surgical resection offers the best opportunity for curing; however, 30–55% will develop recurrence despite curative resection [4]. In these cases, it has been suggested that the presence of a minimal residual disease that remained undetected by imaging or of tumor cells that had already detached and entered the blood stream is associated with recurrence [5]. Additionally, in advanced disease, the numbers of these circulating tumor cells (CTCs) after chemotherapy were reported to reflect subtle metastases and recurrent tumors [6]. Overall, metastasis is the main cause of lung cancer-related death [7].

In contrast to conventional tissue biopsies or cytological preparations, liquid biopsies that contain CTCs and/or circulating tumor DNA (ctDNA) represent a novel approach that illuminates the whole molecular profile of a tumor at the time of sampling [8,9]. Especially in lung cancer, liquid biopsies may outperform tissue biopsies with respect to the tumor’s accessibility at resection. ctDNA is the only approved circulating marker for identifying patients amenable for specific treatments and indeed it has become an indispensable tool for stratifying NSCLC patients [10]. However, the sensitivity of ctDNA platforms can be limited by the small tumor size in early stage disease or after treatment, with minimal residual disease and micrometastasis [11].

In contrast to ctDNA, CTCs are not routinely assessed in the clinics mainly because their scarcity and heterogeneity entail difficult technical requirements. In the age of immunotherapy, however, CTCs may offer potentially useful clinical information at the cellular level, for example, concerning the analysis of the PD-L1 protein expression before treatment as well as during follow-up [12]. Among the plethora of technologies for the analysis of CTCs, the CellSearch assay is the only US Food and Drug Administration (FDA)-approved system for detecting CTCs in cancer patients with metastatic breast, prostate, or colorectal cancer. Several studies have shown the prognostic value of CTC counts in NSCLC [13] and, beyond enumeration, the presence of the EGFR mutation in CTCs [14,15]. Label-free technologies, such as the microfluidic Parsortix™ device, were shown to be more effective in the isolation of EpCAM-negative subpopulations of CTCs [16,17]. Besides the unbiased enrichment of CTCs, this technology is characterized by an outstanding depletion of hematopoietic cells [18], which is key for the molecular analysis of multiple gene transcripts by qPCR.

In recent studies, we evaluated the analysis of CTCs using a combined approach consisting of the microfluidic enrichment by Parsortix™ and of the qPCR-based detection of specific gene transcripts in blood samples from patients with both gynecological malignancies [19] and small-cell lung cancer [20]. In the present study, we applied this combination to blood samples of NSCLC patients and asked whether, in addition to well-established gene markers for epithelial cells (EpCAM and CK19), further markers for ciliated epithelial cells, for the epithelial-to-mesenchymal transition (EMT), and for cancer stem cells (CSC) could indicate the presence of CTCs and may have prognostic relevance in this type of cancer.

## 2. Materials and Methods

### 2.1. Patients and Samples

Blood samples were taken from patients with NSCLC at the Department of Respiratory and Critical Care Medicine, Karl Landsteiner Institute of Lung Research and Pulmonary Oncology, Klinik Floridsdorf, Vienna, Austria. All samples were taken at primary diagnosis or at progression of the disease before the start of a new treatment line. Control blood samples were collected from healthy donors without a history of cancer. All patients and donors had signed a written informed consent. Eighteen milliliters of blood were collected in two Vacuette EDTA tubes (Greiner Bio-One) and processed on the same day in accordance with a recently published protocol [19]. In short, the blood was diluted with an equal volume of phosphate-buffered saline (PBS) and processed using a Parsortix™ microfluidic cassette (Angle plc.) with a critical step size of 6.5µm at 99 mbar pressure. After the separation was completed, the captured cells were harvested and split into two equal parts. For subsequent molecular analysis, one part was immediately lysed by adding 350 µL of RLT lysis buffer (Qiagen). The lysates were stored at −80 °C until RNA extraction. The second half was transferred onto poly-lysine-coated glass slides. After drying, the slides were stored at −20 °C. The study was approved by the Ethics Committee of the Medical University of Vienna, Austria (EK366/2003 and EK2266/2018).

### 2.2. Spiking Experiments

NSCLC lines PC−9 and NCI-H1975 were grown in RPMI 1640 (Invitrogen) supplemented with 10% fetal bovine serum (Invitrogen) and 1% penicillin-streptomycin-gentomycin (Invitrogen) in a humidified atmosphere at 37 °C and 5% CO_2_. At about 70% confluence, the cells were trypsinized and stained with CellTrace Violet (Invitrogen) according to the manufacturer’s protocol. The cell size was assessed using the LUNA-II Cell Counter (Logos Biosystems). Subsequently, 100 stained cells were added manually to an 18 mL control blood sample, which was then processed using the Parsortix™ technology as described above. The efficiency of the Parsortix™ technology to capture NSCLC cells was assessed in duplicate spiking experiments (biological replicates) for each cell line. After the separation was completed, the separation cassette was manually screened for fluorescent cells using a microscope (Olympus BX50) in order to assess the microfluidic capture efficiency. Then, the captured cells were harvested, split into two equal parts (technical replicates), and lysed for subsequent gene expression analysis. Thus, for each cell line, four lysates containing tumor cells were available.

For each cell line, a different donor blood was used and each donor blood was processed in the same way as the spiked sample, albeit with the difference that just one lysate was used for the subsequent gene expression analysis.

### 2.3. Gene Expression Analysis

Total RNA was extracted from the cell lysates using the RNeasy Micro Kit (Qiagen) without DNase treatment. The total amount of RNA was converted into cDNA using the SuperScript VILO Mastermix (Invitrogen). Following a gene-specific pre-amplification, qPCR was done in duplicates in a 10 µL total reaction volume using TaqMan™ UniversalMastermix II and exon spanning TaqMan™ assays (EpCAM, BPIFA1, FAM83A, PTHLH, ERBB3, TWIST1, NANOG, PROM1, MET, UCHL1, TERT, CDH5, and GRP; Life Technologies). The qPCR was performed on the ViiA7 Real-Time PCR System with standard thermal cycling parameters (50 °C for 2 min; 95 °C for 10 min followed by 40 cycles at 95 °C for 15 s and 60 °C for 1 min). A qPCR specific for CK19 was performed at 65 °C annealing/extension with forward and reverse primers that corresponded to published primer sequences and with a FAM-labeled hydrolysis probe (5′-TgTCCTgCAgATCgACAACgCCC-3′) [21]. Raw data were analyzed using the ViiA7 Software v1.1 (Applied Biosystems, Waltham MA, US) with the automatic threshold setting and baseline correction. If the fluorescent signal did not reach the threshold in both duplicate reactions, the sample was regarded as negative for that respective transcript.

### 2.4. Calculation of the Cut-Off Threshold Values

For every marker showing a gene expression background in the healthy donor samples, a cut-off threshold value was calculated by adding the twofold standard deviation to the mean Ct (cycle threshold) value of these “false-positive” control samples [22]. A patient sample was then assigned positive if the Ct value of the respective gene marker was beyond that threshold value. For markers showing no background gene expression, the threshold value was set at the Ct value of 40.0. To evaluate the prognostic significance of each marker, the “optimal” cut point for the Ct value was determined using the function “surv_cutpoint” from the R-package Survminer (version 0.4.2), providing a value of a cut point that corresponds to the most significant relation with overall survival [23]. Afterwards, these threshold values were designated as “diagnostic cut-off” and “prognostic cut-off”. The prognostic cut-off thresholds were calculated for the samples taken at primary diagnosis and at disease progression separately.

### 2.5. Statistical Analysis

The capture rates of PC−9 and NCI_H1975-spiked samples were compared using the unpaired *t*-test. To assess whether the gene expression levels of “false-positive” healthy donor samples and the patient samples were different, a one-way ANOVA was performed. The Pearson’s chi-square test was used to assess the relationship between the presence of each gene transcript beyond the diagnostic and prognostic cut-off as well as the time of blood drawing.

Overall survival (OS) was defined as the period of time in months between blood draw and either death or the last date the patient was seen alive. Kaplan–Meier survival analyses and log-rank tests were used to compare survival outcomes [21]. The Cox proportional hazards regression model was used to determine univariate hazards ratios (HR) for OS [7]. The statistical analysis was performed with R version 4.1.0 and GraphPad Prism version 9.1.2. The level of significance was set at *p* < 0.05.

## 3. Results

### 3.1. Patients and Samples

The characteristics of 118 patients with a histopathological-confirmed diagnosis of NSCLC are shown in Table 1. The mean age was 66.4 years and the distribution of gender was almost equal. The median pack years of the current and former smokers was 55 (range of 30 to 120). The patients were followed for a median of seven (range of 0 to 15) months. The TNM stage was only documented in 38 (32%) cases and thus not included in Table 1. The blood samples were taken at primary diagnosis in 56.8% of the cases and in 32.8% at progression of the disease. In 10.2% of the cases, the time of sampling was not documented.

### 3.2. Spiking Experiments

**Figure 1:**
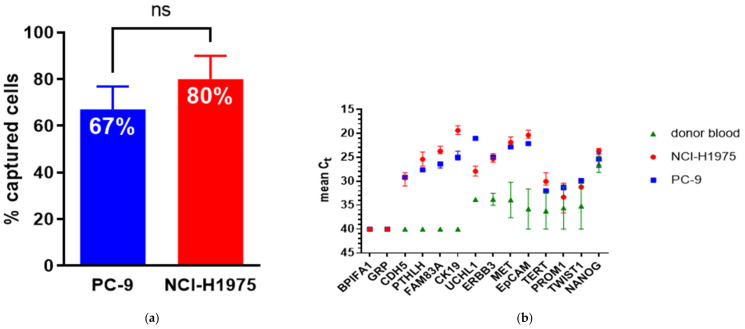
(**a**) Mean rate of PC−9 and H1975 NSCLC tumor cells captured in the Parsortix™ microfluidic separation cassette with a critical step size of 6.5 µm. The lines indicate the range of the capture rates observed in duplicate spiking experiments for each cell line (biological replicate); (**b**) the gene expression analysis of the selected gene markers in Parsortix™-enriched blood samples after a target-specific pre-amplification step. The mean Ct values are derived from the two healthy donor blood samples and for each cell line from the two biological and technical replicates. A Ct value of 40 was inserted to visualize undetectable gene expression by qPCR. Lines indicate the range of Ct values within the replicates.

The efficiency of the microfluidic Parsortix™ system for capturing established NSCLC cell lines in a separation cassette, with a critical gap size of 6.5 µm, is shown in Figure 1a. PC−9 tumor cells with an average size of 13.3 µm were captured at a mean rate of 67% (range of 60–74%) and the larger NCI-H1975 cells (average diameter of 17.4 µm) at a mean of 80% (range of 70–90%). However, the unpaired *t*-test did not indicate a significant difference of the respective capture rates.

The gene expression levels of the selected markers were assessed in all spiked and the corresponding healthy donor blood samples after a gene-specific pre-amplification step. For each cell line, the molecular analysis was done in both biological replicates; furthermore, the analysis of the technical replicates from each biological replicate allowed us to evaluate the performance of the molecular analysis itself as well. Figure 1b depicts the mean Ct values resulting from all replicates containing PC−9 and NCI-H1975 tumor cells and from both replicates of the two healthy donors. As shown in Figure 1b, CDH5, CK19, PTHLH, and FAM83A were detected by qPCR in the spiked samples but not in the respective unspiked donor blood. GRP and BPIFA1 were detected neither in the spiked nor unspiked samples, whereas MET, ERBB3, UCHL1, EpCAM, TERT, PROM1, TWIST1, and NANOG were detected in both, albeit at different gene expression levels.

### 3.3. Lung Cancer Markers in Controls and NSCLC Blood Samples

**Figure 2:**
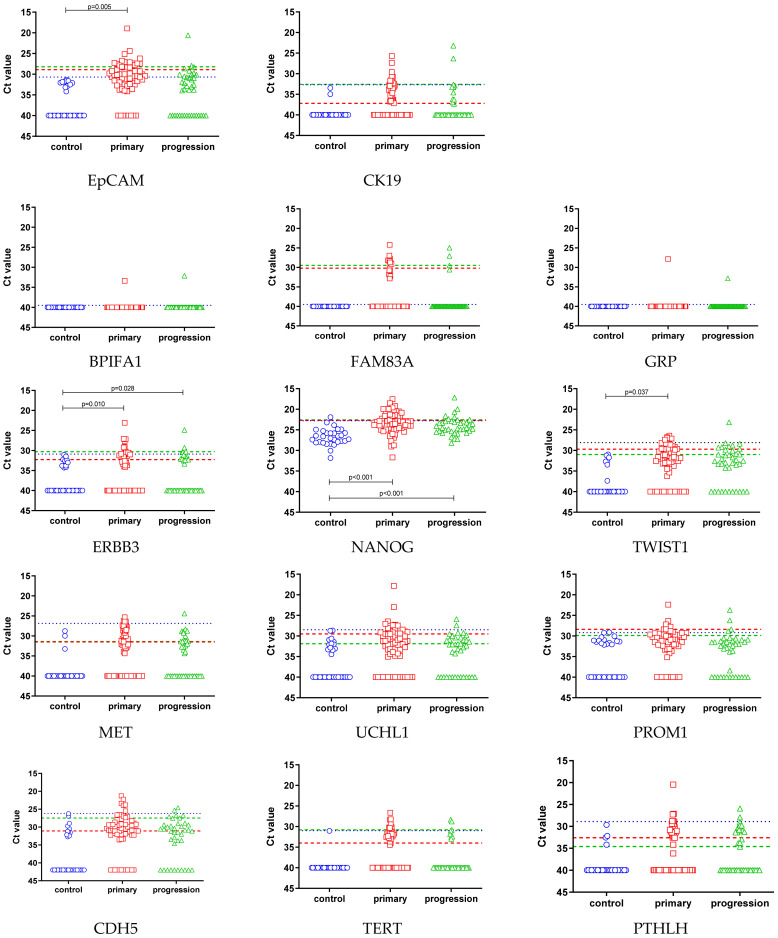
Mean Ct values of all the selected gene markers in the healthy donor blood samples (*n* = 30) and in the NSCLC patient samples taken at both primary diagnosis (*n* = 67) and progression (*n* = 39). Absent gene expression levels are indicated by symbols corresponding to a Ct value of 40. Significantly different gene expression levels in healthy donor blood samples and each group of patient samples are indicated, and the respective *p*-value (ANOVA) is given. The diagnostic threshold levels are indicated by a dotted blue line. The prognostic thresholds of the primary and progression group are indicated by a red and green dashed line, respectively.

The transcript levels of the selected markers were evaluated further in the enriched blood samples from 30 healthy donors and 118 NSCLC patients (Figure 2). The absence of the FAM83A, GRP, and BPIFA1 transcripts, as already indicated in the single unspiked donor blood, was confirmed in this larger set of control samples. All other transcripts were detected in the healthy donor blood samples at varying levels. To assess whether the gene expression levels in these “false-positive” donor samples and the patient samples were different, we performed a one-way ANOVA. ERBB3 and NANOG gene expression levels were significantly higher in both the blood samples taken at primary diagnosis and at progression than in the controls, while increased levels of EpCAM and TWIST1 were observed at primary diagnosis only (Figure 2). For TERT, the statistical test was not performed because just a single donor blood sample was positive.

**Figure 3:**
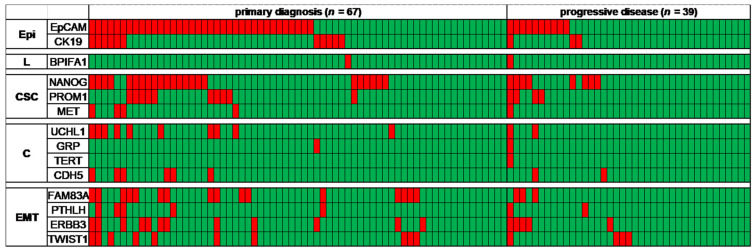
Heat map for transcript-specifics for epithelial cells (Epi), for ciliated epithelial cells in the lung (L), for the epithelial-to-mesenchymal transition (EMT), for cancer stem cells (CSC), and for other cancer-related transcripts (C). Red squares indicate gene expression beyond the calculated diagnostic threshold level per tested sample.

In order to identify patient blood samples with gene expression levels beyond the background in healthy donors, a diagnostic threshold value was calculated to infer the possible presence of tumor cells from a gene expression above that value. Overall, in 85/118 (72.0%) of the patient samples and in just 2/30 (6.7%) of the healthy donor samples, at least one gene marker was detected beyond that threshold, with significantly more positive samples taken at diagnosis than at recurrence (82.1% vs. 53.8%, chi^2^-test *p* = 0.002). The positivity rates of all transcripts are shown in Table 2 and Figure 3. The same threshold value was applied to the 30 healthy control samples to estimate the specificity of the approach (Table 2).

### 3.4. CTC-Related Markers and Patient Outcome

After stratifying the patients by the previously calculated diagnostic threshold value into two groups, NANOG and PROM1 were the only gene markers associated with worse outcomes, regardless of whether they were detected in blood samples taken at primary diagnosis or at disease progression (Supplementary Materials Table S1). In contrast, FAM83A and PTHLH were associated with poor OS only in samples taken at progression (Supplementary Materials Table S1).

**Figure 4:**
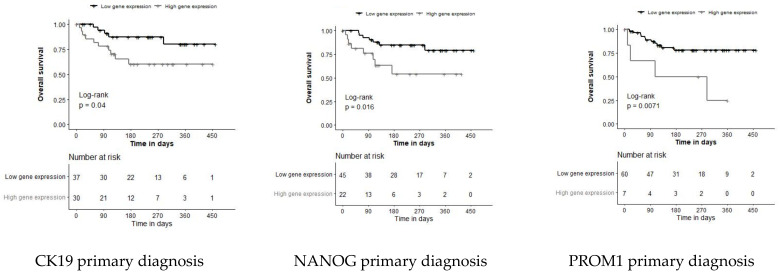

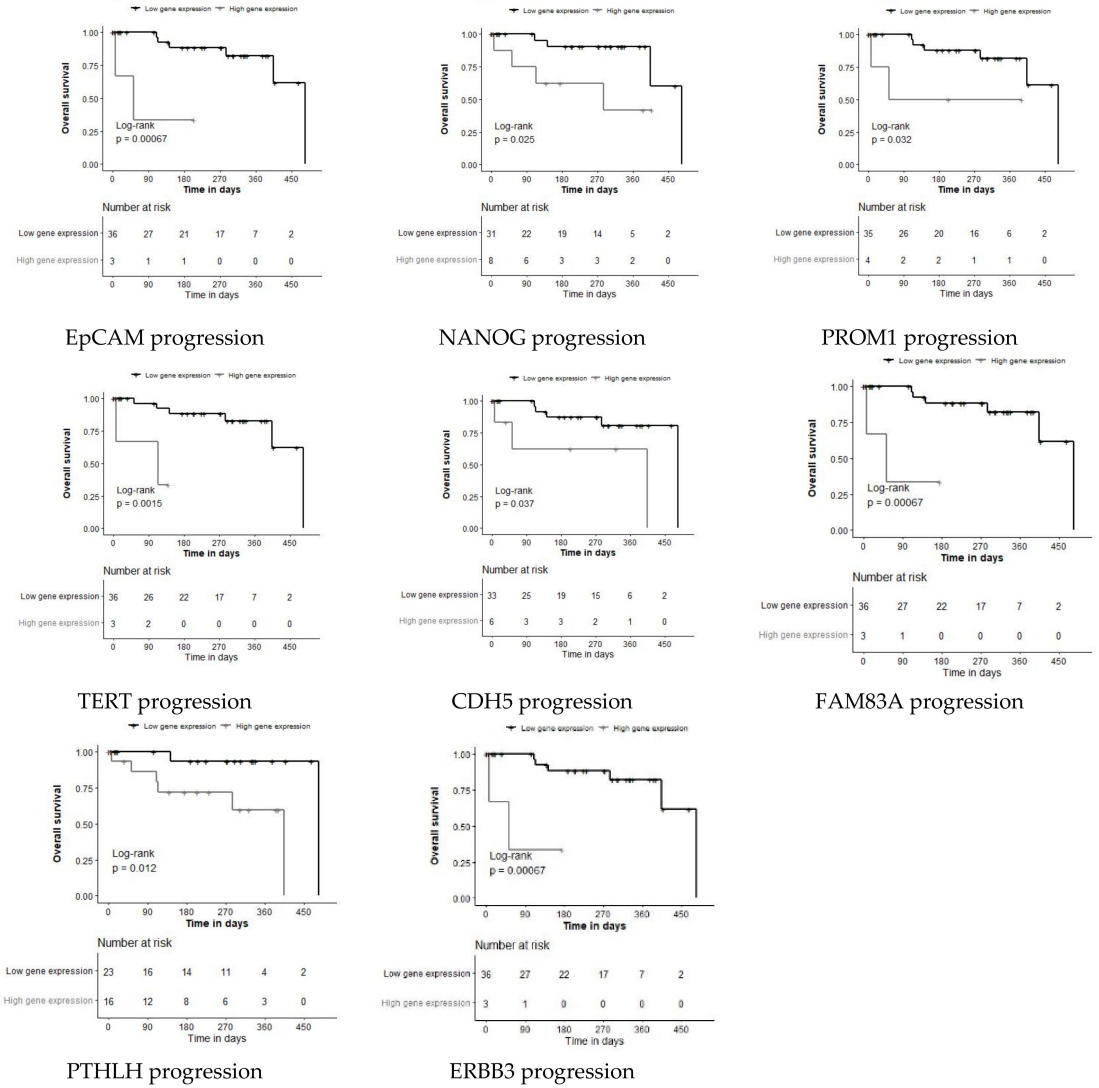
Overall survival plots of patients with univariate significant gene expression levels stratified by the prognostic threshold determined using the R-package Survminer, providing a value of a cut point that corresponds to the most significant relation with overall survival at primary diagnosis and at disease progression. *p*-values correspond to log-rank tests.

However, replacing the diagnostic threshold value by the “optimal” cut point added CK19 as a further prognostic marker at primary diagnosis (Figure 4). In samples taken at disease progression, EpCAM, ERBB3, TERT, and CDH5 showed prognostic relevance as well (Figure 4). While the diagnostic and prognostic thresholds were almost identical for PROM1 and NANOG, the prognostic threshold of EpCAM was more stringent than the diagnostic threshold. Thus, the percentage of EpCAM-positive samples at diagnosis decreased from 53.7% to 26.9% and from 25.6% to 7.7% at progression, at the same specificity, with none of the healthy donor samples being false-positives. The same phenomenon was observed with CDH5 when a more stringent cut-off resulted in a significantly higher risk of death in CDH-positive already progressive patients. The difference between the respective diagnostic and prognostic cut-off values of ERBB3 and TERT was minimal; nevertheless, the prognostic cut-off was able to identify these markers as being prognostic in patients with progressive disease. Table 3 shows the prevalence of positive samples and the impact on OS as assessed by univariate Cox regression analysis after stratifying the patients by that “optimal” prognostic cut-off value.

## 4. Discussion

In the present study, we applied a recently established workflow for the molecular detection of CTCs enriched with the microfluidic Parsortix™ system [19] to blood samples taken from NSCLC patients at primary diagnosis or at progression of the disease. The enriched cells were analyzed at the molecular level for the presence of epithelial cell lineage-specific markers (EpCAM and CK19), EMT-related markers (FAM83A, PTHLH, ERBB3, and TWIST1), CSC-related markers (NANOG, PROM1, and MET), the lung-specific marker BPIFA1, and the general cancer-related markers UCHL1 and GRP.

We detected EpCAM transcript levels above the diagnostic threshold in 53.7% of the blood samples at primary diagnosis. At the start of a new treatment course, significantly fewer EpCAM-positive samples were observed, which, at first glance, seems contradictory because one might suggest a higher tumor load at progression. However, NSCLC patients are closely monitored and, after an initial response, even a slight increase of tumor burden not necessarily associated with an increase of CTC numbers may prompt a new treatment line. In any case, only after applying a more stringent prognostic threshold value, EpCAM gene expression levels were significantly related with OS in patients with progressive disease. Similarly, the application of the R-package Survminer revealed the prognostic significance of CK19 at primary diagnosis as well as of other cancer-related (CDH5 and TERT) and EMT markers (FAM83A, PTHLH, and ERBB3) at disease progression. Most importantly, our study shows the significant prognostic relevance of the CSC-specific transcripts NANOG and PROM1 both at primary diagnosis and at disease progression.

The observed prevalence of epithelial CTCs enriched with the Parsortix™ system is in line with Papadaki et al. who found epithelial CTCs by IF-staining following enrichment by the same microfluidic system in 60% of NSCLC patients before treatment [24]. In addition, our recovery rates for the NCI-H1975 cell line correspond to the values reported by those authors as well as to those evaluated by a multi-center ring trial testing several CTC-enrichment technologies including the Parsortix™ system [25].

Noticeably, the lack of the prognostic significance of epithelial CTCs in our study and in Papadaki’s study is in contrast to the significant association of CTCs, with OS reported by the largest clinical study of CellSearch-CTCs in NSCLC to date [26]. Considering the fact that in most studies, CTC counts are converted into categorical variables by grouping patients into two or more groups, the importance of choosing the optimal cut point for categorization cannot be overstated. What applies for CTC numbers [26,27] applies even more for gene expression levels.

In parallel to the molecular analysis using qPCR, we performed an IF-staining of the enriched cells in nine representative patients (Supplementary Materials Table S2). Here, CTCs were assessed by positive staining of EpCAM and/or cytokeratins (Supplementary Materials Figures S1 and S2). In comparing the binary outputs of either methodology, we observed a considerable agreement of qPCR and IF, although, in the case of the cytokeratins, we did not target the same protein/transcripts by IF (CKs 4/5/6/8/10/13/18) and qPCR (CK19). In our recent study comparing IF and qPCR for the detection of CTCs in ovarian cancer patients, we achieved just moderate agreement by using a panel of antibodies for IF (including EpCAM) and cyclophilin C transcripts for the qPCR-based detection of CTCs [28]. In addition to other technical constraints, in that study, the CTCs were enriched using a density gradient centrifugation [29], which still leaves an enormous amount of residual leukocytes contributing to false-positive results in the control group. All this suggests that, indeed, an utmost depletion of contaminating cells is key for the precise detection of CTCs by qPCR at least for most of the putative markers.

In the past, it has been argued that IF, and more specifically the FDA-cleared CellSearch technology, can provide a numeric value of CTC counts, which, indeed, has been shown to be prognostic in various cancer types. Nevertheless, the advantages of an additional molecular characterization using qPCR are manifold, particularly regarding the openness to perform high-throughput and multiplex analyses [30], and thus allowing for the testing of promising biomarkers in the expanding field of precision oncotherapy in order to select the most promising therapeutic strategy for the individual patient based upon the gene expression profile of isolated CTCs [31]. Recent examples include—without any claim of completeness—our own study proving the molecular detection of DLL3 (Delta-like Canonical Notch Ligand 3), a target of Rova-T in CTCs from small-cell lung cancer patients [20] and other drugs targeting DLL3 that are in clinical trials currently; a further study showing the association of AR-v7 (androgen receptor splice variant 7) in CTCs and the treatment failure of abiraterone and enzalutomide in castration-resistant prostate cancer patients [32]; the CirCe T-DM1 trial demonstrating the actionability of HER2-amplified CTCs in HER2-negative metastatic breast cancer [33]; and ovarian cancer studies evidencing the ERCC1 in CTCs as a prognostic and predictive biomarker [34] for resistance to platinum-based chemotherapy [35,36].

NANOG is a homeobox domain transcription factor, which is a key regulator of embryonic development and cellular reprogramming [37], and is broadly expressed in various cancers [38,39,40]. Its overexpression was shown to be associated with poor prognosis in NSCLC [34,41,42] and, moreover, predicted poor response to platinum treatment [41] in this cancer type as well as in ovarian cancer [42]. In CTCs, increased NANOG expression was observed in head and neck squamous cell carcinoma patients responding to treatment with nivolumab [43]; however, in untreated patients, overexpression was not associated with the outcome [44]. The authors explain this contradictory finding by stating that blood samples from patients responding to nivolumab may be enriched with CSC-like CTCs not diminished by treatment [43]. A recent study showed the association of NANOG-positive CTCs with the recurrence of hepatocellular carcinoma [45]. Furthermore, NANOG may be clinically relevant in monitoring patients treated with a CSC inhibitor, such as Napabucasin (BBI608), whose strong anti-CSC effect has already been demonstrated in vitro and in vivo in a broad range of cancer types [46].

Besides NANOG, the cell surface molecule PROM1 (also referred to as CD133), a widely accepted CSC marker [47], proved to be prognostic in our study. This finding is in line with Nel et al. who reported an association of mesenchymal CTCs, an increased ratio of CD133+ stem cell-like CTCs to epithelial CTCs, and poor treatment response [48].

An unexpected result in our study was the low frequency of BPIFA1-positive samples. BPIFA1 (also referred to as PLUNC or LUNX) is reported to be highly specific for ciliated alveolar epithelial cells. For this reason and because our own previous whole transcriptome studies of healthy donor blood samples indicated the absence of BPIFA1 transcripts in healthy blood [49], we assumed that BPIFA1 would be an ideal marker for lung cancer CTCs. Nonetheless, in the present study, BPIFA1 was observed in just a single sample at diagnosis and recurrence. This discrepancy to Katseli et al., who reported LUNX-positive CTCs in about one third of the patients [50], and to Li et al., with an even two-fold prevalence [51], could be explained by different methodological approaches to enrich the CTCs and to detect BPIFA1-specific transcripts. The same authors report the presence of PTHLH in 65% of the NSCLC patients [50], while in our study, PTHLH was observed in less than 10% of the patients.

## 5. Conclusions

In summary, the present study highlights the prognostic value of the CSC-related NANOG and PROM1 transcripts in CTCs enriched by a label-free device from blood samples taken from patients with primary or progressive disease. Our findings underline the relevance of CTCs other than ctDNA and may strengthen the role of CTCs as an additional tool for the clinical management of NSCLC patients.

## Figures and Tables

**Table 1:** Characteristics of the 118 NSCLC study patients.

Characteristics	*n* (%)
Age (years):	
Mean (median)	66.4 (66.0)
Range	46.0 - 89.0
Gender:	
Male	60 (50.4)
Female	59 (49.6)
Tobacco abuse:	
Current smokers	35 (29.4)
Former smokers	36 (30.3)
Never smokers	15 (12.6)
Unknown	33 (27.7)
Outcome at study completion:	
Dead	25 (21.0)
Alive	94 (79.0)
Blood draw for CTCs:	
At primary diagnosis	67 (56.8)
At progression/recurrence	39 (32.8)
Unknown	12 (10.2)

CTCs circulating tumor cells.

**Table 2:** Prevalence of gene expression levels beyond the diagnostic threshold in 30 healthy donor blood samples and 118 NSCLC patients. The absolute and relative numbers of positive findings is given for the total study population and stratified by the stage of disease at blood draw (primary diagnosis and progression). The chi^2^-test was performed to examine the relation between marker positivity and stage of disease at time of blood draw (primary diagnosis vs. progressive disease).

	Threshold	Healthy	NSCLC			
		All Donors (*n* = 30)	All Patients (*n* = 118)	Primary Diagnosis (*n* = 67)	Progression (*n* = 39)	*p*
Overall		2 (6.7%)	85 (72.0%)	55 (82.1%)	21 (53.8%)	0.002
	Epithelial cell-specific					
EpCAM	30.7	0	54 (45.7%)	36 (53.7%)	10 (25.6%)	0.005
CK19	32.7	0	15 (12.7%)	11 (16.4%)	3 (7.7%)	0.201
	Ciliated epithelial-cell specific					
BPIFA1	40.0	0	2 (1.7%)	1 (1.5%)	1 (2.6%)	0.696
	Cancer stem cell-related					
NANOG	22.8	1 (3.3%)	34 (28.8%)	23 (34.3%)	8 (20.5%)	0.132
PROM1	29.2	1 (3.3%)	16 (13.6%)	10 (14.9%)	4 (10.3%)	0.494
MET	26.9	0	6 (5.1%)	4 (6.0%)	1 (2.6%)	0.425
	Cancer-related					
UCHL1	28.5	0	13 (11.0%)	10 (14.9%)	2 (5.1%)	0.125
GRP	40.0	0	3 (2.5%)	2 (3.0%)	1 (2.6%)	0.883
TERT	31.0	0	37 (31.4%)	9 (13.4%)	4 (10.3%)	0.631
CDH5	26.2	0	8 (6.8%)	6 (9.0%)	2 (5.1%)	0.472
	EMT-related					
FAM83A	40.0	0	23 (19.5%)	16 (23.4%)	4 (10.3%)	0.084
PTHLH	28.9	0	8 (6.8%)	5 (7.5%)	2 (5.1%)	0.641
ERBB3	30.9	0	18 (15.3%)	12 (17.9%)	5 (12.8%)	0.491
TWIST	28.1	0	9 (7.6%)	6 (9.0%)	1 (2.6%)	0.201

The most frequently detected transcript was EpCAM, with 53.7% and 25.6% positive samples at diagnosis and recurrence, respectively (chi^2^-test *p* = 0.005, Table 2). A chi-square test of independence was performed to examine the relation between EpCAM positivity and disease stage. qPCR-positive samples at primary diagnosis were not more often EpCAM-positive than the 21 qPCR-positive samples taken at disease progression (*p* = 0.155). Similarly, CSC and EMT-related transcripts were equally abundant at primary diagnosis and at progression of the disease, as was the total number of positive markers above the threshold (primary diagnosis: median 2, range of 1–9 markers; progression: median 1, range of 1–13 markers).

**Table 3:** Prevalence of gene expression levels beyond the prognostic cut-off threshold levels as assessed for samples taken at primary diagnosis and at progression. The absolute and relative numbers of positive findings beyond these thresholds are shown for the healthy donor group and the respective patient group. Univariate Cox hazards for OS are shown for both groups of patients, each stratified by the prognostic cut-off threshold values of the respective gene marker.

	Prognostic Cut-Off at Primary Diagnosis						Prognostic Cut-Off at Disease Progression					
Marker	Threshold	Healthy (*n* = 30)	Patients (*n* = 67)	HR	95% CI	*p*	Threshold	Healthy (*n* = 30)	Patients (*n* = 39)	HR	95% CI	*p*
Epithelial cell-specific												
EpCAM	28.9	0	18 (26.9%)	2.58	0.81–8.27	0.056	28.2	0	3 (7.7%)	9.39	0.21–422.74	<0.001
CK19	37.2	2	30 (44.8%)	2.92	1.04–8.19	0.040	32.6	0	2 (5.1%)	2.03	0.12–33.09	0.480
Ciliated epithelial cell-specific												
BPIFA1	NA.						NA					
Cancer stem cell-related												
NANOG	22.7	0	22 (32.8%)	3.21	1.02–10.14	0.016	22.6	0	8 (20.5%)	4.17	0.72–24.14	0.025
PROM1	28.4	0	7 (10.4%)	4.23	0.65–27.56	0.007	28.9	0	4 (10.2%)	4.77	0.29–78.94	0.032
MET	31.5	2	23 (34.3%)	1.84	0.62–5.44	0.230	31.4	2	12 (30.1%)	3.47	0.49–24.54	0.059
Cancer-related												
UCHL1	29.5	2	18 (26.9%)	0.43	0.14–1.34	0.250	31.9	6	15 (38.5%)	3.13	0.78–12.51	0.110
GRP	NA.						NA					
TERT	34.0	1	24 (35.8%)	1.62	0.56–4.67	0.350	30.8	0	3 (7.7%)	8.32	0.23–304.78	0.002
CDH5	31.1	5	39 (58.2%)	0.56	0.20–1.59	0.260	27.5	2	6 (15.3%)	4.00	0.51–30.96	0.037
EMT-related												
FAM83A	30.2	0	9 (13.4%)	0.37	0.09–1.45	0.310	29.5	0	3 (7.7%)	9.39	0.21–422.74	<0.001
PTHLH	32.6	3	22 (32.8%)	2.48	0.83–7.36	0.070	34.6	4	16 (41.0%)	5.63	1.31–24.22	0.012
ERBB3	32.3	3	22 (32.8%)	0.41	0.14–1.16	0.150	30.3	0	3 (7.7%)	9.39	0.21–422.74	<0.001
TWIST	29.7	0	18 (26.9%)	1.75	0.56–5.42	0.280	31.0	1	12 (30.1%)	2.59	0.44–15.22	0.160

HR hazard ratio and CI confidence interval. NA threshold values not assessed due to small sample number.

## Data Availability

The data presented in the study are available upon request from the corresponding author.

## Supplementary Materials

The following are available online at https://www.mdpi.com/article/10.3390/jpm11111225/s1, Supplementary Table S1: Univariate Cox hazards for the OS of the NSCLC patients stratified by the diagnostic cut-off threshold values of each gene marker.; Supplementary Figure S1: Image gallery showing CTCs in patient #9 (left panel) and a healthy donor blood sample containing both leukocytes (green arrow) and spiked tumor cells (red arrow, right panel). Tumor cells were defined as cytokeratin and/or EpCAM-positive (red signal), DAPI-positive (blue signal), and CD45-negative cells (absence of green signal); Supplementary Table S2: Blood samples of nine patients were assessed using both qPCR and IF staining. The absolute counts of EpCAM and/or CK-positive and CD45-negative CTCs are shown, as well as the gene expression of EpCAM, CK19, and NANOG beyond (1) or below (0) the diagnostic threshold value, and the total number of gene markers beyond the same threshold; and Supplementary Figure S2: Swimmer plot of patients with epithelial CTCs detected by IF-staining and the presence or absence of NANOG gene expression in the same sample. Head-arrows indicate that the patient was still living at study completion and black squares indicate that the patient has died. Circles indicate the absolute CTC numbers by IF. The length of the bars indicate the overall survival after the blood draw, with checkered bars for patients with NANOG gene expression levels beyond the threshold value.
