# Supplementary material for: Cancer Stem Cell-Like Circulating Tumor Cells Are Prognostic in Non-Small Cell Lung Cancer
Source: J Pers Med. 2021 Nov 18;11(11):1225. doi: 10.3390/jpm11111225 (PMC8620949; doi:10.3390/jpm11111225)
Supplement: Supplementary file 1 [file jpm-11-01225-s001.zip › jpm-1432580-supplementary.pdf]

Survival analysis using the diagnostic cut-off threshold

Supplementary Table S1. Univariate Cox hazards for the OS of the NSCLC patients stratified by the diagnostic cut-off threshold values of each gene marker.

		Primary Diagnosis (n=67)			Disease Progression (n=39)		
**Marker**	**Threshold**	**HR**	**95% CI**	**p**	**HR**	**95% CI**	**p**
EpCAM	30.7	1.21	0.44-3.36	0.713	1.28	0.23-7.06	0.754
CK19	32.7	0.77	0.20-3.00	0.732	2.02	0.12-33.08	0.481
BPIFA1	40.0	NA			NA		
NANOG	22.8	2.87	0.94-8.81	0.032	4.17	0.72-24.13	0.025
PROM1	29.2	3.37	0.75-15.23	0.018	4.77	0.29-78.96	0.030
MET	26.9	0.34	0.05-2.34	0.272	NA		
UCHL1	28.5	0.39	0.10-1.61	0.351	5.29	0.07-401.4	0.078
GRP	40.0	NA			NA		
TERT	31.0	1.55	0.54-4.43	0.396	2.05	0.39-10.80	0.150
CDH5	26.2	0.53	0.11-2.54	0.531	1.54	0.13-18.53	0.675
FAM83A	40.0	1.26	0.37-4.28	0.693	5.52	0.28-109.9	0.016
PTHLH	28.9	0.71	0.12-4.06	0.734	9.29	0.21-410.8	<0.001
ERBB3	30.9	0.67	0.18-2.46	0.596	3.13	0.30-32.82	0.133
TWIST	28.1	0.68	0.12-3.86	0.712	NA		

HR hazard ratio and CI confidence interval. NA threshold values not assessed due to small sample number

Immunofluorescent staining (IF)

IF was performed on parallel cytological preparations of the Parsortix-enriched blood samples from nine representative patients. The glass slides containing the enriched cells were blocked with Lab Vision™ Ultra V Block (Thermo Scientific, USA) for 5 min. Then, the primary antibodies labeled with a fluorophore were applied in the appropriate concentration in Antibody Diluent Dako REAL™ Antibody Diluent (anti-CD45-AF488, Biolegend, 1:200; anti-cytokeratin-AF594, Biolegend, 1:800 recognizing human cytokeratin 4, 5, 6, 8, 10, 13, and 18; and anti-EpCAM-AF555, CellSignalling 1:50). After incubation for 1 h at room temperature in the dark, the slides were washed three times in PBS. After, counterstaining the cell nuclei with DAPI (Thermo Fisher Scientific) for 3 min and three final washings, the slides were mounted with Fluoromount-G™ (Southern Biotech). CTCs mounted on glass slides were visualized and scanned with the CellCelector microscope (Automated Lab Solutions, Jena, Germany). CTCs were defined as cytokeratin and/or EpCAM-positive, DAPI-positive, and CD45-negative cells, and counted from scanned images (Supplementary Figure S1).

We observed a substantial agreement between the results obtained by IF and qPCR considering the corresponding epithelial markers EpCAM and CK19 only (chi-square 89%, Cohen's κ=0.61). Worse OS was not merely associated with the presence of epithelial CTCs detected by IF: for example, patient 2 died 18 days after the blood draw and epithelial CTCs were not detected by IF (Supplementary Table S2). All other patients with extremely short survival times were rather NANOG-positive by qPCR than had epithelial CTCs (Supplementary Figure S2).

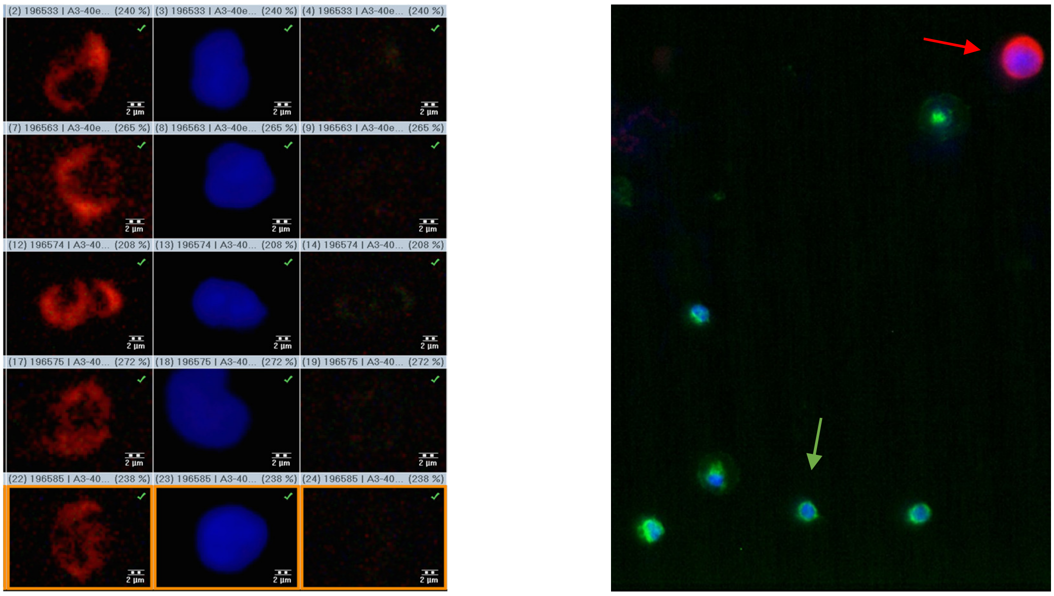

Patient CTCs Healthy donor blood spiked with tumor cells

Supplementary Figure S1. Image gallery showing CTCs in patient #9 (left panel) and a healthy donor blood sample containing both leukocytes (green arrow) and spiked tumor cells (red arrow, right panel). Tumor cells were defined as cytokeratin and/or EpCAM-positive (red signal), DAPI-positive (blue signal), and CD45-negative cells (absence of green signal).

Supplementary Table S2. Blood samples of nine patients were assessed using both qPCR and IF-staining. The absolute counts of EpCAM and/or CK-positive and CD45-negative CTCs are shown, as well as the gene expression of EpCAM, CK19, and NANOG beyond (1) or below (0) the diagnostic threshold value, and the total number of gene markers beyond the same threshold.

Patient ID	ClinicalCharacteristics			CTCs			
	**Blood draw**	**Outcome**	**OS (days)**	**Cell counts**	EpCAM	CK19	NANOG
1	progression	alive	104	0	0	0	0
2	primary	dead	18	0	1	0	1
3	primary	dead	424	10	0	1	0
4	primary	alive	30	12	0	1	0
5	primary	alive	303	17	1	1	0
6	primary	alive	286	18	0	1	0
7	primary	alive	321	48	1	1	0
8	primary	dead	32	133	1	1	1
9	progression	dead	8	155	1	1	1

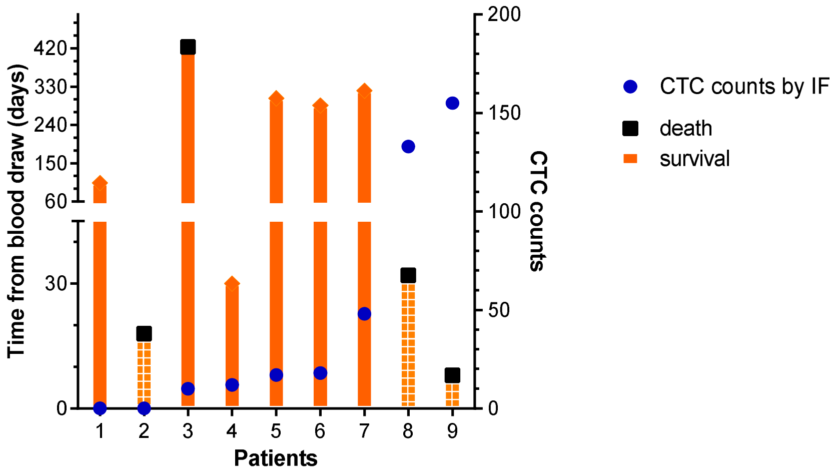

Supplementary Figure S2. Swimmer plot of patients with epithelial CTCs detected by IF-staining and the presence or absence of NANOG gene expression in the same sample. Head-arrows indicate that the patient was still living at study completion and black squares indicate that the patient has died. Circles indicate the absolute CTCs numbers by IF. The length of the bars indicate the overall survival after the blood draw, with checkered bars for patients with NANOG gene expression levels beyond the threshold value.
